# Mechanisms of action of NME metastasis suppressors – a family affair

**DOI:** 10.1007/s10555-023-10118-x

**Published:** 2023-06-24

**Authors:** Céline Prunier, Philippe Chavrier, Mathieu Boissan

**Affiliations:** 1 Sorbonne Université, INSERM UMR_S 938, Centre de Recherche Saint-Antoine, CRSA, Paris, France; 2 Actin and Membrane Dynamics Laboratory, Institut Curie – Research Center, CNRS UMR144, PSL Research University, Paris, France; 3 Laboratoire de Biochimie Endocrinienne Et Oncologique, Oncobiologie Cellulaire Et Moléculaire, APHP, Hôpitaux Universitaires Pitié-Salpêtrière-Charles Foix, Paris, France

**Keywords:** Metastasis, Nucleoside diphosphate kinase, Dynamin, GTP, Channeling

## Abstract

Metastatic progression is regulated by metastasis promoter and suppressor genes. *NME1*, the prototypic and first described metastasis suppressor gene, encodes a nucleoside diphosphate kinase (NDPK) involved in nucleotide metabolism; two related family members, NME2 and NME4, are also reported as metastasis suppressors. These proteins physically interact with members of the GTPase dynamin family, which have key functions in membrane fission and fusion reactions necessary for endocytosis and mitochondrial dynamics. Evidence supports a model in which NDPKs provide GTP to dynamins to maintain a high local GTP concentration for optimal dynamin function. NME1 and NME2 are cytosolic enzymes that provide GTP to dynamins at the plasma membrane, which drive endocytosis, suggesting that these NMEs are necessary to attenuate signaling by receptors on the cell surface. Disruption of NDPK activity in NME-deficient tumors may thus drive metastasis by prolonging signaling. NME4 is a mitochondrial enzyme that interacts with the dynamin OPA1 at the mitochondria inner membrane to drive inner membrane fusion and maintain a fused mitochondrial network. This function is consistent with the current view that mitochondrial fusion inhibits the metastatic potential of tumor cells whereas mitochondrial fission promotes metastasis progression. The roles of NME family members in dynamin-mediated endocytosis and mitochondrial dynamics and the intimate link between these processes and metastasis provide a new framework to understand the metastasis suppressor functions of NME proteins.

## ***NME*** genes as suppressors of metastasis

Metastasis is the main cause of death in cancer patients. During metastatic disease, complex factors involving the tumor cells and their microenvironment promote tumor dissemination from the primary site, tumor cell survival in the bloodstream, extravasation and colonization of a secondary site [1]. Breakdown of intercellular adhesion in the tumor epithelium and acquisition of invasive and migratory traits allow epithelial tumor cells to breach the basement membrane and degrade the interstitial – mostly – type I collagen in the stromal extracellular matrix (ECM). These processes, referred collectively to as the epithelial–mesenchymal transition (EMT), are critical events in tumor progression [2, 3]. As in tumorigenesis, metastasis is regulated both positively (by promoters) and negatively (by suppressors). Metastasis suppressor genes – unlike tumor suppressors – inhibit metastasis without affecting growth of the primary tumor [4]. Genetic alterations in these genes – i.e. loss of heterozygosity, spontaneous mutations and polymorphisms – are rare in primary tumors, thus metastasis suppressor genes are probably inactivated by epigenetic mechanisms during metastatic dissemination.

The human genome is estimated to contain ~ 30 genes that encode proteins with metastasis suppressor activity [4–8]. The first example of such a metastasis suppressor gene, *NME1* [9]*,* has been characterized extensively and is the subject of numerous reviews [10–14]. The role of the related *NME2* gene in the context of metastasis suppression is much less well documented and remains controversial [15–18]. *NME3* has not yet been found to have metastasis suppressor activity, whereas *NME4* was described very recently as a new metastasis suppressor gene, the first one in mitochondria [19].

*NME* genes encode highly conserved multifunctional proteins, some of which are nucleoside diphosphate kinases (NDPKs). NDPKs catalyze the transfer of a γ-phosphate group from nucleoside triphosphates (mainly ATP) to nucleoside diphosphates (mainly GDP) [20–22]. These proteins have many important physiological functions in bioenergetics, cytoskeleton and membrane dynamics, signal transduction, metabolism, and development and they are also involved in pathological processes, including metastasis, through mechanisms that are beginning to be deciphered [23–26]. In mammals, the *NME* gene family comprises ten members that can be divided into two phylogenetic groups [23, 27]. The group I genes (*NME1*–*NME4*) encode proteins that are 58–88% identical to each other and have only one NDPK domain (Table 1). The four NME1 to NME4 proteins are ubiquitous and their catalytic activities have similar kinetics [28]. NME1, NME2 and NME3 are mainly located in the cytosol and at the plasma membrane, whereas NME4 is located exclusively in mitochondria [23, 24, 29, 30]. NME1 and NME2 assemble *in vitro* and *in vivo* into stable, catalytically active homohexamers and/or heterohexamers [31, 32]. Formation of such oligomers may contribute to regulating their subcellular localization and/or cellular functions. NME1 and NME2 are the two most abundantly expressed and are thought to be responsible for at least 80% of the cell’s NDPK activity. The phylogenetic group II genes encode more divergent proteins that are only 22–44% identical to each other and to the group I enzymes. Group II NMEs contain one or several full-length or truncated NDPK domains [27]. Despite these domains, none has a demonstrated catalytic activity. In addition, these proteins contain other domains, such as the Dpy-30, DM10, or thioredoxin domains, which may regulate the localization and/or the function of the proteins, possibly by modulating their interactions with various partners. Most group II NME proteins are found only in ciliated cells; the exception is NME6, which is ubiquitous and is located in mitochondria [33].

**Table 1:**
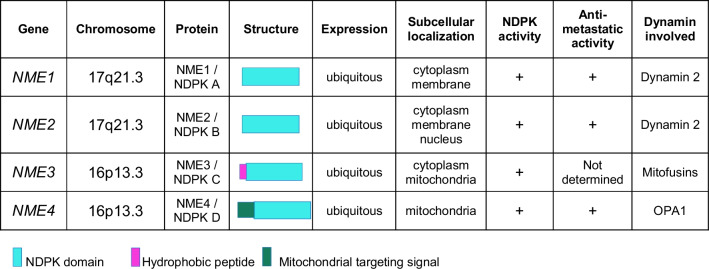
The group I NME family members

### *NME1*, a prototypic metastasis suppressor

Two original discoveries established *NME1* as a metastasis suppressor. First, up-regulation of *NME1* in several metastatic tumor cell lines from multiple histological types, including melanoma, breast, colon, lung, liver, ovary, prostate, and oral carcinoma cell lines, reduced their metastatic potential both in experimental and spontaneous mouse models of metastasis [34–42]. Second, the incidence of lung metastases increased significantly in *NME1* knockout mice prone to develop hepatocellular carcinoma [43]. In addition, numerous studies analyzing human patients with melanoma or epithelial tumors of the breast, liver, colon or ovary, found that loss of *NME1* expression correlated with a greater risk of metastasis and a poorer clinical prognosis [10, 37, 44–49]. By contrast, in neuroblastoma and in hematological malignancies, *NME1* overexpression correlated with metastasis dissemination and unfavorable outcome [50–52]. These conflicting reports of both positive and negative correlation of *NME1* expression with metastasis depending on the tumor type, are highly suggestive of context-specific mechanisms.

Overexpression of *NME1* in invasive tumor cell lines that have low levels of endogenous *NME1* expression reduces the migratory and invasive potential of the cells. This is observed in cell lines derived from melanoma and from breast, colon, lung, liver, ovarian, prostate and oral carcinoma [42, 53–59]. By contrast, silencing *NME1* in non-invasive tumor cell lines that express high NME1 levels, results in a migratory and invasive phenotype in melanoma, glioblastoma, and carcinoma cells [60–64]. Silencing *NME1* in epithelial tumor cells results in an intermediate phenotype with both epithelial and mesenchymal features, refered to as a ‘partial’ EMT [65]. Indeed, *NME1*-depleted cells express both epithelial and mesenchymal markers, indicating that they do not transition fully to a mesenchymal state. Nevertheless, this ‘partial’ EMT phenotype is more metastatic than the fully mesenchymal state [66, 67], providing an explanation for the high metastatic potential of NME1-deficient tumor cells.

In human cancer samples, there is an inverse correlation between *NME1* levels and the expression of well-known EMT markers [65]. Consistent with this, NME1 is downregulated or absent at the invasive front of clinical samples of hepatocellular carcinoma and colon cancers whereas it is robustly present in the center of the tumors [60]. In addition, the ability of *NME1* to influence tumor invasion *in vivo* has been demonstrated in a xenograft model in which human breast tumor cells were injected into the primary duct of the mammary gland in immunodeficient mice [68, 69]. In this model, tumor cells form intraductal tumors that recapitulate the transition from carcinoma *in situ* to invasive breast carcinoma [68, 70]. Gene-editing inactivation of *NME1* in the injected cells accelerated the development of invasive lesions [61].

### *NME2*, a controversial metastasis suppressor

The anti-metastatic activity of *NME2* has been demonstrated mainly in breast, lung and oral squamous carcinoma, and in melanoma [15, 40, 71–73]. Moreover, analysis of gene expression datasets from breast, colon, lung and ovarian tumors revealed that there is significantly less *NME2* expression in metastatic tumors than in non-metastatic tumors [74].

*NME2* has been reported to be an inhibitor of cell motility and invasion [18, 55, 57, 75, 76], suggesting that it might have a similar function to *NME1* during tumor progression as an inhibitor of metastasis. In contrast to these earlier studies, however, our work rather suggests that *NME2* is not a suppressor of EMT and tumor invasion [60, 61, 65, 77]. Indeed, loss of *NME2,* unlike *NME1*, does not impair the invasive capacity of tumor cells or the transition from *in situ* to invasive breast carcinoma in the intraductal xenograft model [61]. Importantly, inactivation of either *NME1* or *NME2* expression did not alter the protein level of the other isoform [61], suggesting that there is no compensatory effect when one isoform is deficient.

NME1 and NME2 isoforms are 88% identical in their amino acid sequence, however, sixteen out of eighteen non-identical amino acids are located at the peripheral surface of the hexamer [78]. Thus, NME1 and NME2 homohexamers may interact with different partners and may have distinct cellular functions. Indeed, there is evidence that NME1 and NME2 have more specific partners than partners in common [78]. Furthermore, the relative abundance of NME1 and NME2 proteins and the stoichiometry of NME1–NME2 heterohexamers may provide additional specific regulation and function.

In addition to specific binding partners and regulation of function of NME1 and NME2, the two proteins may function at distinct times in tumorigenesis. We hypothesise that that NME1 and NME2 function as NDPKs at early stages of tumorigenesis, providing nucleoside triphosphates to sustain the high proliferation of tumor cells. At later stages, however, NME1 might become the only isoform controlling EMT, invasion and metastasis. Consistent with this hypothesis, cells induced to proliferate have high levels of NME1 [60, 79–83] and cells overexpressing *NME2* proliferate faster than control cells in culture [73]. Accordingly, NME1 and NME2 are overexpressed early in the primary tumor and only NME1 expression is reduced or repressed at later stages in colorectal and hepatic tumors that have undergone metastasis [43, 60, 84]. Additionally, in breast cancer, we found a similar up-regulation of NME1 in ductal carcinoma *in situ* when compared to the surrounding non-malignant tissues, whereas NME1 levels were significantly reduced in invasive tumor foci and in microinvasive carcinoma buds extending beyond the ruptured basement membrane [61]. By contrast, NME2 was upregulated in ductal carcinoma *in situ*, but remained highly expressed in invasive tumors supporting the conclusion that NME2 is not a repressor of invasion.

### *NME4*, a new metastasis suppressor

The *NME4* gene encodes nucleoside diphosphate kinase D (NDPK-D). Mutations in *NME4* that inactivate either the enzymatic activity of NDPK-D or its ability to bind cardiolipin in the mitochondrial inner membrane both induce a strong metastatic phenotype in the cervical carcinoma cell line HeLa and in the breast adenocarcinoma cell line MDA-MB-231 [19], including pronounced cell scattering, loss of intercellular adhesion, increased cell migration in 2D and 3D assays, and increased invasion through a type I collagen matrix. Overexpression of wild-type NDPK-D had the opposite, anti-metastatic effect. Conversely, silencing *NME4* in the breast epithelial carcinoma cell line ZR75-1 reduced cell–cell adhesion and increased migration [19]. The metastasis suppressor activity of *NME4* was most clearly demonstrated in a metastasis assay in mice in which HeLa cells overexpressing wild-type NDPK-D were injected intravenously [19]. Significantly fewer lung metastases were seen in these mice than in mice injected with HeLa cells overexpressing kinase-dead NDPK-D or expressing a low level of wild-type NDPK-D. These effects were specific to the altered function/expression of mitochondrial NME4 and not due to modified expression of NME1 and NME2 [19].

Low levels of *NME4* expression also correlated with high levels of metastasis in various types of cancer in humans. *NME4* expression is lower in hepatocarcinoma-derived cell lines with high metastatic potential than it is in those with low metastatic potential [85]. In human cancer, *NME4* expression correlates negatively with markers of EMT and tumor aggressiveness [19]. In several cohorts of breast cancer patients, expression of *NME4* is negatively associated with markers of mesenchymal cells, the EMT, and tumor invasion, but is positively associated with epithelial markers. In oral cancer, a miRNA that promotes cell migration, invasion and metastasis by inhibiting *NME4* expression, miR-196, is highly expressed and correlates with lymph node metastasis [86]. Consistent with the role of NME4 as a metastasis suppressor in human subjects, low expression of *NME4* is associated with shorter overall survival (i.e. poor prognosis) in patients with breast tumors or with several other tumor types [19].

### Other *NME* genes

Among the other members of the *NME* gene family, there is sparse evidence for their functions as suppressors of metastasis. Human *NME3* and *NME5* might modulate tumor cell motility depending on the cell context [55, 87], but the mechanism(s) are unclear. Also, in one study, overexpression of *NME3* inhibited the metastatic potential of colorectal tumor cells [87]. Further work will be required to pursue the characterization of these and other *NME* family genes roles in cancer.

## The dynamin connection

In this section we will review the cellular and molecular mechanisms whereby NME proteins suppress metastasis; in particular, we will detail a mechanism related to NME’s interplay with dynamin GTPases.

Classical dynamins are GTPase motor proteins that are required for endocytosis in all eukaryotic cells [88]. They are responsible for the scission of endocytic vesicles from the plasma membrane during clathrin-mediated endocytosis as well as in some clathrin-independent endocytic pathways [89, 90]. The interaction of NME proteins with dynamins is conserved across species from the nematode *Caenorhabditis elegans*, to the fruit fly *Drosophila melanogaster*, mice and humans [26, 91–94]. NMEs in dynamin-mediated endocytosis might suppress metastasis by inhibiting cell migration and cell invasion, and maintaining cell–cell adhesion, all of which are important for EMT and metastasis processes [3, 95].

### NME/dynamin interplay in cell migration and chemotactism

The first evidence for a functional link between NME proteins and dynamin came from a study in *Drosophila* [91], that showed that Awd, the counterpart of mammalian NME1 and NME2 facilitated dynamin-mediated neurotransmitter uptake at neuromuscular junctions in the fly. Further studies reported a key implication of Awd function in endocytosis during cell migration [96, 97]. In cooperation with the *Drosophila* homolog of dynamin, Shibire, Awd inhibits cell migration by promoting endocytosis of chemotactic receptors including the receptors for FGF and PDGF/VEGF, from the surface of migrating tracheal cells during tracheogenesis and of migrating border cells during oogenesis [96–101]. Loss of *awd* in these two cell types decreases endocytosis, leading to up-regulation of the receptors on the cell surface and increasing migration. By contrast, overexpression of *awd*, increases the endocytosis rate of receptors from the cell surface, so decreasing cell migration. The severity of the *awd* phenotype is exacerbated in a *shibire* mutant background whereas overexpression of *awd* can revert the phenotype associated with a dominant-negative *shibire* mutation. Likewise in mammalian cells, NME1 mediates endocytosis of the FGF receptor, FGFR1, induced by expression of the von Hippel-Lindau (VHL) protein and prevents cell migration [102]. Interestingly, a loss-of-function mutant in *VHL* [103] resembles the tracheal phenotype in the *awd* mutants [96], suggesting that the functional relationship between VHL and NME is evolutionary conserved and is important during development. In addition, mammalian tumor cell lines overexpressing *NME1* have increased endocytosis of the EGF receptor and also migrate less than the control cells, and both the increased endocytosis and suppression of migration are blocked by inhibitors of dynamin [57]. In the nematode *Caenorhabditis elegans*, the NDPK homolog of NME1 and NME2, NDK-1, also influences migration of distal tip cells [104]. Although the underlying mechanism is unknown, the genes encoding *C. elegans* NDK-1 and dynamin, DYN-1, interact genetically [104]. Additionally, in a genome-wide RNAi screen for genes involved in membrane trafficking, knockdown of NDK-1 caused failure of receptor-mediated endocytosis [105]. Thus, it is possible that NDK-1 regulates the amount of a chemotactic receptor on the surface of the distal tip cells, in the same way as it does in *Drosophila* and in mammals.

Together, the evidence discussed above indicates that NME proteins facilitate endocytosis of surface receptors and possibly other proteins, altering their availability to transduce migration signals, which, in turn, can suppress cell migration and chemotactism.

### NME/dynamin interplay during cell invasion

Regulation of endocytosis rate by NME proteins may also influence the ability of tumor cells to invade surrounding tissues by clearing membrane-bound proteases from the cell surface. Transmembrane membrane type 1 metalloproteinase (MT1-MMP) is instrumental during cancer progression by mediating proteolytic breaching of tissue barriers, basement membrane and interstitial stromal type I collagen. MT1-MMP is cleared from the cell surface by dynamin-dependent clathrin-mediated endocytosis and is found in clathrin-coated pits associated with dynamin [106–108]. Overexpression of *NME1* in breast tumor cells was found to increase MT1-MMP endocytosis resulting in removal of the protease from the cell surface, whereas silencing of *NME1* decreases MT1-MMP uptake [61]. Collectively, these data indicate that NME1 controls the endocytic clearance and surface exposure of MT1-MMP in human breast cancer. As a consequence, loss of NME1 function enhances both matrix degradation and the invasive potential of breast tumor cells *in vitro*. At the mechanistic level, MT1-MMP, NME1 and dynamin, interact in clathrin-coated vesicles at the plasma membrane. Loss of *NME2*, by contrast, has no such effect. Consistent with a role for NME1 in endocytosis of MT1-MMP, in human hepatoma and colorectal tumor cell lines in which *NME1* is silenced, expression of a mutant *MT1-MMP* deleted of its catalytic domain inhibits invasion [60]. Conversely, overexpression of proteolytically active invasion-promoting MT1-MMP increases invasion by cells in which *NME1* is silenced. Thus, NME1 enhances endocytosis of MT1-MMP, so suppressing cell invasion.

### NME/dynamin interplay in cell adhesion

Another way by which the function of NME proteins in dynamin-mediated endocytosis may suppress metastasis is by promoting cell adhesion. Evidence for this mechanism, again, came first from studies of the *awd* mutation in the *Drosophila* homolog of *NME1* and *NME2.* During oogenesis in mutant *awd* larvae, adherens junction components, including E-cadherin, β-catenin and α-spectrin, in follicle epithelial cells are mislocalized in the oocyte, disrupting the integrity of the epithelium structure, whereas *awd* overexpression promotes the turnover of these components by controlling endocytosis [109]. Consistent with this, *awd* mutations cause developmental defects in the imaginal discs, the sac-like epithelial structures in *Drosophila *larvae from which legs, antennae and wings develop in the adult fly [110, 111]. A kinase-dead *awd* mutation failed to rescue the *awd* mutant phenotype in contrast to the wild-type *awd* [112], indicating that Awd kinase activity is essential for cell adhesion during *Drosophila *development.

Consistent with a role for Awd in cell adhesion, dynamin is also necessary to maintain epithelial integrity [113, 114]. In epithelial tissues with a *shibire* mutation, E-cadherin accumulates in the cytoplasm and adherens junction stability is disrupted, indicating that E-cadherin endocytosis is regulated in epithelial tissues and necessary to maintain epithelium integrity [113–116]. Similarly, in *C. elegans* embryos, dynamin-mediated endocytosis is crucial to maintain cell polarity [117]. Moreover, in non-invasive hepatoma and colon tumor epithelial cell lines, silencing *NME1* reduces the amount of E-cadherin on the cell surface, correlating with reduced cell–cell adhesion [60]. Also in mammalian epithelial cells, NME1 promotes dynamin-mediated endocytosis of E-cadherin [118].

NME proteins may also regulate cell adhesion to the substratum by modifying the endocytosis of integrin receptors. ICAP-1, an adaptor protein for clathrin-dependent endocytosis of integrins, recruits NME1 and NME2 close to integrins, the clathrin adaptor protein complex, AP2, and dynamin at clathrin-coated pits to ensure integrin turnover at focal adhesions and regulate integrin signaling and cell adhesion [119–121].

This abundant evidence that NME proteins are involved in clearing from the cell surface various receptors with key functions in cell–cell adhesion and cell adhesion to the matrix by promoting their dynamin-mediated endocytosis, so inhibiting cell migration and invasion, likely explains the strong metastasis suppressor activity of NME1.

### Control of mitochondrial dynamics by NME family proteins

Additional members of the dynamin superfamily act elsewhere in eukaryotic cells to mediate membrane fission and fusion [122, 123]. Three human mitochondrial dynamin-like proteins, dynamin-related protein 1 (DRP1), optic atrophy protein 1 (OPA1) and mitofusin (MFN), could be implicated in the metastasis suppressor function of mitochondrial NME4 and possibly NME3.

Changes in mitochondria structure and function are potent determinants of EMT and metastasis [124–126]. In particular, fragmentation (or fission) of the mitochondrial network facilitates invasion and migration of tumor cells, whereas mitochondrial fusion is rather inhibitory [127]. Generally, metastatic tumor cells express low levels of the fusogenic protein, MFN, as compared to non-metastatic cells, while they express higher levels of the pro-fission protein, DRP1 [128–131]. In addition, activation of DRP1 [132] or MFN silencing [128] increase the metastatic potential, whereas silencing or pharmacological inhibition of DRP1 or MFN overexpression reduce cell migration and metastasis [128, 129, 133, 134].

The two isoforms of mitofusin, MFN1 and MFN2, are integral membrane proteins of the mitochondrial outer membrane that mediate fusion [122, 123]. NME3 is similarly localized at the outer membrane depending on its N-terminal region [93], and it is known to interact with MFN1 and MFN2 [135]. Silencing of *NME3* increases fragmentation of mitochondria [136], suggesting that NME3 enhances outer membrane fusion mediated by mitofusins. Strikingly, expression of NME3 was shown to rescue mitochondrial fusion and elongation in *NME3*-silenced cells irrespective of its NDPK activity [135]. Further studies will be necessary to elucidate exactly how NME3 functions in mitochondrial fusion and whether this mechanism can contribute to suppress metastasis. In addition, it should be noticed that the NDPK ortholog DYNAMO1 (dynamin-based ring motive-force organizer 1) of mammalian NME3 locally generates GTP for the optimal activity of DRP1 during the division of mitochondria in the red alga, *Cyanidioschyson merolae* [137]. However, the relevance of these data regarding the role of NME3 in metastasis dissemination is currently unknown.

In addition, the dynamin-like protein, OPA1, mediates the fusion between the inner membranes of mitochondria [122, 123]. NME4, is also located in the intermembrane space and is bound to the mitochondrial inner membrane through cardiolipin, an abundant phospholipid in the inner membrane [138]. Silencing of *NME4* alters mitochondrial morphology by producing fragmented, swollen and ‘blebby’ mitochondria reminiscent of those produced upon defective mitochondrial fusion [93]. Depletion of *NME4* phenocopies the effect of *OPA1* loss-of-function on mitochondria morphology. Moreover, NME4 forms a complex with OPA1 on the mitochondrial inner membrane, facilitated by the binding of both proteins to cardiolipin [138–140]. The effect of NME4 binding to OPA1 is not fully understood, however NME4 increases GTP-loading onto OPA1 [93], suggesting that NME4 promotes OPA1-mediated fusion activity. The functions of NME3 and MFN at the outer membrane and NME4 and OPA1 at the inner membrane, respectively, likely contribute to maintaining a fused mitochondrial network important for preventing the EMT and metastasis.

### Molecular mechanisms related to dynamin function displayed by NME family proteins

**Fig. 1:**
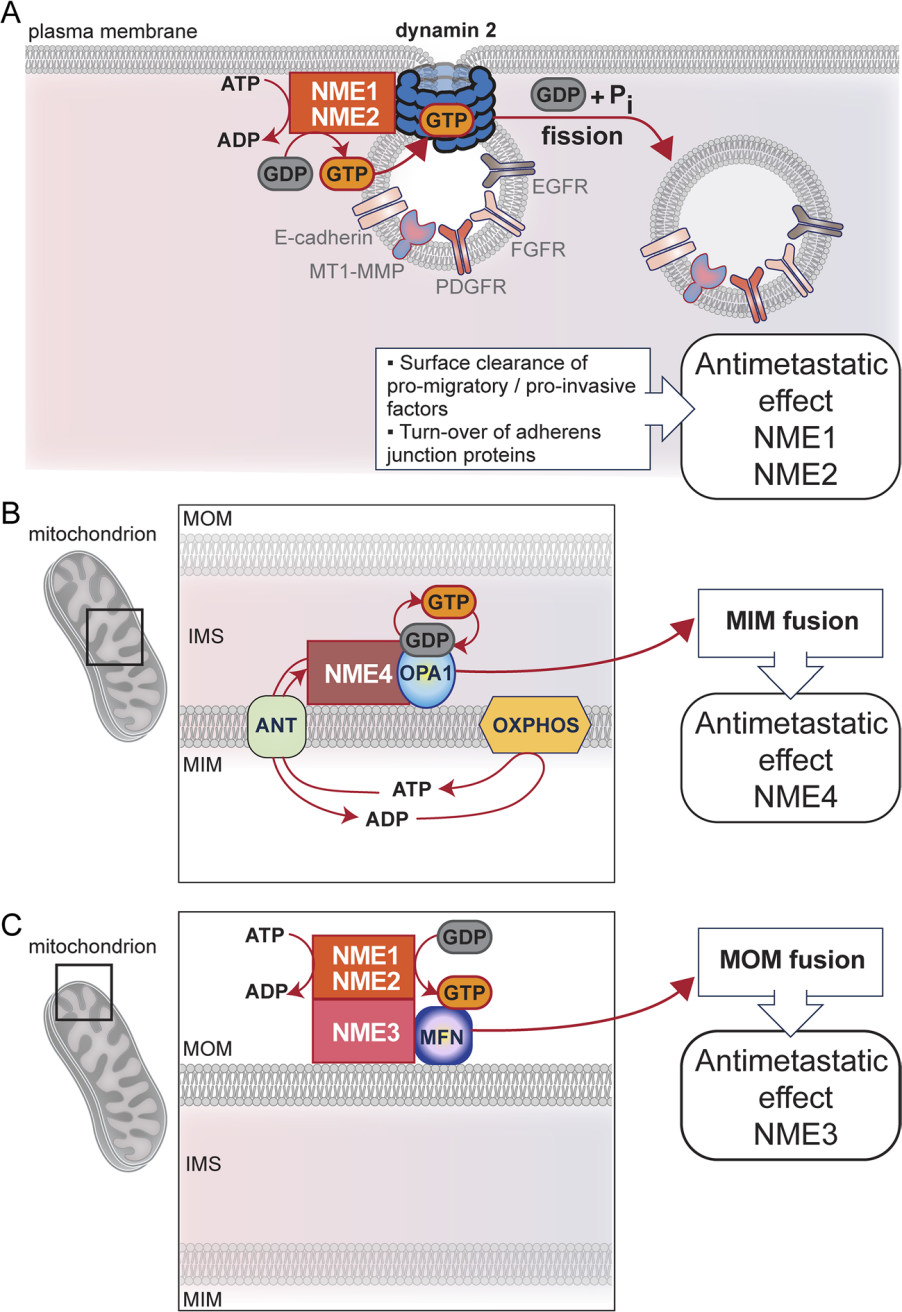
Mechanisms of antimetastatic action of group I NME family members. **A.** The cytosolic NDPKs NME1 and NME2 are recruited to the plasma membrane by their physical interaction with dynamin 2. They generate GTP locally from GDP and ATP and channel GTP to dynamin 2 to optimise dynamin’s activity, which is necessary for fission and endocytosis. The resulting removal of pro-migratory/pro-invasive factors such as MT1-MMP, PDGFR, FGFR and EGFR from the cell surface and turnover of adherens junction proteins such as E-cadherin explain the antimetastatic effects of NME1 and NME2. **B.** The mitochondrial NDPK NME4 binds the dynamin-related protein OPA1 at the mitochondrial inner membrane to provide GTP for OPA1, which permits mitochondrial inner membrane fusion. This fusion process inhibits metastasis, thus explaining the antimetastatic activity of NME4. **C.** The localization of NME3 at the mitochondrial surface, where the dynamin-related pro-fusion proteins mitofusins act, suggests that this NDPK might assist mitofusins during mitochondrial outer membrane fusion. NME3 might recruit the cytosolic NDPKs NME1 and NME2 to the mitochondrial surface to produce GTP for mitofusins and promote mitochondrial outer membrane fusion. This fusion process also inhibits metastasis, thus this mechanism may explain the antimetastatic effect of NME3. PDGFR: platelet-derived growth factor receptor, FGFR: fibroblast growth factor receptor, EGFR: epidermal growth factor receptor, MOM: mitochondrial outer membrane, IMS: intermembrane space, MIM: mitochondrial inner membrane, ANT: adenylate translocase, OXPHOS: oxidative phosphorylation, MFN: mitofusins

Members of the dynamin superfamily are evolutionarily conserved membrane-remodeling GTPases involved in both membrane fission and fusion reactions. However, unlike myosin and kinesin motor proteins that use ATP to produce forces, dynamins hydrolyse GTP. In addition, dynamins are very sensitive to intracellular GTP concentration due to their remarkably low affinity for GTP and high intrinsic GTPase activity, resulting in rapid GTP hydrolysis and GDP–GTP exchange, which is enhanced by dynamin oligomerization [90, 93, 141]. Thus, vigourous replenishment of GTP is necessary to sustain cellular activity of dynamin. Unlike for myosins and kinesins, which are fueled by high cytosolic ATP concentration, the lower concentrations of GTP are not sufficient to maintain high rate of GTP loading and GTP hydrolysis by dynamins [142]. Thus, a mechanism of GTP channeling achieved by enzymes that synthesise GTP in close proximity to dynamins is required to secure a high GTP/GDP ratio and favour GTP hydrolysis. The strong NDPK activity of NME proteins [143], together with their high affinity for GDP [144], are ideal to maintain a high local concentration of GTP required for dynamin function. Indeed, several evidence support a model in which NDPKs physically interact with members of the dynamin superfamily to maintain high local GTP concentration for optimal dynamin function in membrane remodeling [94]. NME1 and NME2 fuel cytoplasmic endocytic dynamins at plasma membrane clathrin-coated pits to drive endocytosis (Fig. 1A). In addition, NME1 and NME2 may facilitate the oligomerization of dynamin, which is necessary for membrane fission [57]. Thus, local production and channeling of GTP to endocytic dynamins and stimulation of dynamin oligomerization by NME1 and NME2 contribute to stimulate dynamin’s function in endocytosis. NME4 has the same function on OPA1 by fueling GTP on it at the mitochondria inner membrane to drive inner membrane fusion (Fig. 1B). The molecular mechanism underlying NME3 function on mitofusins must be different to this GTP channeling mechanism as a kinase-dead mutant of NME3 can rescue mitochondrial fusion in *NME3*-silenced cells. One possibility is that NME3 recruits cytosolic NDPKs, NME1 and/or NME2, to the mitochondrial surface by forming hetero-oligomers. In this scenario, NME1/NME2 recruited by NME3 would channel GTP to mitofusins to promote outer membrane fusion (Fig. 1C). If the molecular mechanisms of action of NME proteins on dynamin superfamily proteins remain to be defined precisely, convergence of subcellular localisations of NME proteins and their dynamin superfamily counterparts, acting as a team is emerging as a new, interesting perspective to explain the antimetastatic activity of NME proteins (Table 1).

## Cytoskeleton regulation by NME family proteins

Additionally mechanisms may explain how NME1 and, in some cases, NME2 can potently suppress cell migration and metastasis. On the one hand, NME proteins inhibit Rho GTPase signaling, by sequestering nucleotide exchange factors necessary for Rho activation in the control of cell cytoskeleton dynamics. Indeed, NME1 sequesters Tiam1 and Dbl-1, the nucleotide exchange factors for Rac1 and cdc42, respectively [145–147], whereas NME2 sequesters Lbc, the nucleotide exchange factor for RhoA [148]. The independent identifications of three nucleotide exchange factors as NME-interacting proteins suggest that the Rho GTPase signaling may be biologically relevant. On the other hand, NME1 may suppress metastasis, by reducing transcription of the *EDG2* gene, which is involved in metastasis and connected with Rho–ROCK regulation of cell motility [38, 63]. Third, the anti-metastatic function of NME1 may be mediated partially by its ability to inhibit the activity of the actin-severing protein gelsolin [149]. A proteomics study identified NME1 as a binding partner of gelsolin [149]. NME1 inhibited the actin depolymerizing activity of gelsolin, antagonized gelsolin-stimulated tumor cell migration *in vitro*, and attenuated its pro-metastatic activity in an *in vivo* model of breast tumor metastasis. Fourth, NME1 is reported to inhibit cell migration by phosphorylating the light chain of the cytoplasmic motor protein myosin [150]. Finally, accumulating evidence suggests that NME1, and often also NME2, interact with and affect the functions of various components and regulators of the cytoskeleton, including acting-binding proteins, intermediate filaments and attachment sites for the cytoskeleton (adherens junctions, desmosomes, and focal adhesions) in cells from a variety of organisms and tissues and during the course of development, suggesting this association is conserved through evolution and may serve an essential function [26].

## Conclusion

Over the last three decades, extensive analyses of the NME/NDPK family revealed the multifaceted roles of these conserved proteins in cellular pathophysiology and uncovered the underlying molecular mechanisms. The role of the NME/NDPK family in membrane remodeling and nucleotide channeling has become widely recognized as an essential feature of the mechanism of action of several NME family members, including NME1, NME2, NME4, and potentially NME3. Here, the classical NDPK model as a main source of GTP is extended to direct fueling of GTP to GTP-dependent dynamin family proteins through protein/protein interaction. This conclusion is supported by experimental evidence obtained in different species and model systems, including the fruit fly D. *melanogaster*, nematode C. *elegans*, mouse and human, indicating an evolutionary conserved mechanism of membrane remodeling controlled by dynamin-NME/NDPK protein interplay. GTP fueling to cytosolic dynamins (through cytosolic NME1 and NME2), which promotes endocytosis of cell surface receptors and cargoes with key function in cell–cell adhesion and cell adhesion to the matrix with, direct impact on cell migration and invasion, likely explains the strong metastasis suppressor potential of NME1 and its alternatively, NME2. GTP fueling to mitochondrial dynamin-related OPA1 (by mitochondrial NME4) promotes mitochondrial inner membrane fusion, a process inhibitory to migration and invasion of tumor cells. The localization of NME3 at the outer mitochondrial membrane, where the fusogenic dynamin-like protein, mitofusin, is recruited to, mediates mitochondrial outer membrane fusion, suggesting that NME3 might likewise participates in outer membrane dynamics, although through a mechanism different of GTP channeling. These emerging roles of NME family members in dynamin-mediated endocytosis and mitochondrial dynamics provide a new framework to explain the antimetastatic activities of NME proteins, which may open new routes to novel therapeutics.
